# Does the gender of emergency physicians have an impact on the prehospital care of psychiatric emergencies? a retrospective cohort analysis

**DOI:** 10.1186/s12873-024-01118-3

**Published:** 2024-10-24

**Authors:** Benedikt Schick, Benjamin Mayer, Bettina Jungwirth, Eberhard Barth, Claus-Martin Muth, Christine Eimer, Celine Schwarzer, Carlos Schönfeldt-Lecuona

**Affiliations:** 1 Department of Anesthesiology and Intensive Care Medicine, University Hospital Ulm, Albert-Einstein- Allee 23, 89081 Ulm, Germany; 2 Institute of Epidemiology and Medical Biometry, Ulm University, Schwabstraße 13, 89075 Ulm, Germany; 3 Department of Anesthesiology and Intensive Care Medicine, University Medical Center Schleswig- Holstein, Campus Kiel, Arnold-Heller Str. 3, 24105 Kiel, Germany; 4 Department of Psychiatry and Psychotherapy III, University Hospital Ulm, Leimgrubenweg 12–14, 89075 Ulm, Germany

**Keywords:** Emergency medicine, Emergency therapy, Mental disorders, Primary health care, Psychiatric emergency, Gender

## Abstract

**Background:**

Psychiatric emergencies pose a special challenge for emergency physicians. It is known from other areas of medicine that the influence of a doctor’s gender can have an impact on the type of treatment and quality of patient care. However, this has not yet been investigated in the context of prehospital care in psychiatric emergencies.

**Objectives:**

To identify whether the gender of the prehospital emergency physicians has an influence on the “on-scene” time, treatment strategy and on the potential escalation of interventions for patients with a psychiatric diagnosis.

**Methods:**

A retrospective cohort analysis of emergency missions with a psychiatric diagnosis was performed between January 1, 2015 and December 31, 2021 at the Department of Emergency Medicine, Department of Anesthesiology and Intensive Care Medicine, University Hospital Ulm, Germany.

**Results:**

2882 emergency missions with a psychiatric indication/prehospital psychiatric diagnosis were studied and divided into: intoxication (*n* = 1343, 46.6%), suicidal behavior (*n* = 488, 16.9%), exceptional mental situation (*n* = 282, 9.8%), agitation (*n* = 262, 9.1%), anxiety and panic disorders (*n* = 262, 9.1%) and “psychiatric miscellaneous” (*n* = 245, 8.5%). Inpatient hospitalization occurred in 67.9% (*n* = 1958) of emergency missions. Of these, 20.3% (*n* = 392) were admitted directly to a psychiatric hospital. Male emergency physicians had a slightly longer "on-scene" time for psychiatric emergencies than female emergency physicians (*p* = 0.024). However, the variance in "on-scene" time for all interventions was significantly greater for female emergency physicians than for male emergency physicians (*p* = 0.025). Male emergency physicians were significantly more likely than their female counterparts to administer intravenous hypnotics in prehospital psychiatric emergencies (*p* = 0.001). For psychiatric patients who refused medically indicated inpatient psychiatric admission (“involuntary psychiatric admission”), male and female emergency physicians were equally likely to take the required action (*p* = 0.522). However, male emergency physicians were significantly more likely to administer an intravenous hypnotic to enforce involuntary admission (*p* = 0.009).

**Conclusions:**

Similar to other medical specialties where the influence of physician gender on patient care is certainly relevant, the gender of prehospital emergency physicians also appears to influence their prehospital management strategy in psychiatric emergencies. The influence of gender is sometimes subtle and limited to specific aspects, such as the administration of hypnotics. Prospective study designs are needed to thoroughly investigate the influence of the gender of the prehospital emergency physician on the quality of care in psychiatric emergencies.

**Trial registration:**

The study was approved by the ethics committee of the University Ulm, Trial-Code No. 110/22 and was prospectively registered in the German Clinical Trials Register (DRKS-ID: DRKS00031237). Patient information was not required for retrospective data analysis.

**Supplementary Information:**

The online version contains supplementary material available at 10.1186/s12873-024-01118-3.

## Background

The German emergency medical services system is characterized by a distinct separation between prehospital and inpatient care. Prehospital care is provided primarily by paramedics. They have limited authority to administer medications and perform invasive procedures. Invasive measures include prehospital intubation and chest tube placement. In the case of serious illnesses or unclear medical histories, an prehospital emergency physician is dispatched according to the “rendez-vous” approach, i.e. the physician is brought to the patient [1]. In most cases, they are anesthetists, internal medicine specialists or surgeons. Specialists in psychiatry who serve as prehospital emergency physician are less than 0.5% in the German system [2].The German training concept for prehospital emergency physicians focuses strongly on the treatment of somatic disorders. The prehospital treatment of psychiatric emergencies, although the third most common indication for intervention, is not sufficiently addressed [2, 3]. This is reflected in the uncertainty reported by prehospital emergency physicians and a self-reported skills deficit in the prehospital management of psychiatric emergencies [2, 3]. Existing uncertainty is compounded by the lack of algorithms for standardized prehospital care of these patients. Finally, prehospital emergency physicians also report a lack of motivation to care for psychiatric emergencies [4].

While clear quality criteria and indicators for measuring the quality of medical care have been defined in other areas of emergency medicine, i.e. duration of pre-hospital phase, diagnosis and monitoring, guideline-based patient care, they have been completely lacking in the care of prehospital psychiatric emergencies [5]. For instance, it remains unclear whether the "on-scene" time, defined as a valuable quality indicator in health care research, i.e. the time between the arrival of the emergency physician and the start of transport, can also be used to assess the quality of medical care in psychiatric emergencies [6]. The generally required basics of a patient-oriented and thus empathic doctor-patient relationship play a special role in the context of prehospital relationship building with psychiatric emergency patients. The emergency physician often has only a few minutes to make a decision about the acuity of the symptoms and thus about the necessary therapy, even against the patient’s will. According to Mercer and Reynolds, “empathy as a multidimensional construct is the ability of the physician to understand the current situation, the patient’s perspective and feelings, communicate what they have understood, check for consistency, act accordingly, and support the patient in a helpful and therapeutic way [7].”

In this context, escalation of measures, i.e., the use of restraints or physical coercion, should be considered a failure of a de-escalation strategy. Although measures against the will of the mentally ill patient cannot always be avoided, the nature of the chosen approach should be such that physical and psychological interventions in the integrity of the patient’s autonomy are kept to a minimum [2, 8]. However, it is conceivable that gender differences between emergency physicians may influence their prehospital behavior in dealing with psychiatric emergencies and, consequently, the duration of emergency care. At present, it is not possible to derive any implications for the evaluation of the quality of emergency care.

Particularly in the field of medical communication, as an integral part of the multidimensional definition of empathy, there are a number of studies that have examined the influence of the physician gender on the physician-patient relationship [9–14]. Female physicians appear to have more empathetic communication skills than male physicians [9, 14]. They seem to understand and verbalize the patient’s current situation and needs better than their male colleagues. These are elementary medical skills, especially in the prehospital setting, where there is little time to establish a trusting relationship with the psychiatric emergency patient. However, they are probably rarely applied in this form in everyday life. The demands on communication skills when dealing with psychiatric patients are high. Active listening and a sense of cultural nuance are among the most important [15]. In this context, excellent communication and interpersonal skills are also defined as core competencies of the emergency physician [16–20]. Various training programs attempt to teach these communication skills in the context of the unique challenges of emergency medicine. However, it is unclear how gender differences affect both communication and knowledge transfer in prehospital emergency medicine.

To reduce this evidence gap and to elicit future studies on this topic, we analyzed the management of prehospital psychiatric emergencies by male and female prehospital emergency physicians using an open-ended approach. The results will be used to suggest ways of improving prehospital emergency care of psychiatric emergencies. Furthermore, the aim is to raise awareness of possible gender differences within emergency medicine.

## Study objectives

The primary objectives of the study were to assess, whether the gender of the prehospital emergency physician:

- has an influence on the duration of prehospital “on-scene” time.
- has an influence on the treatment and potential escalation of interventions for patients with a suspected psychiatric diagnosis.

The daily practice indicates that patients with psychiatric disorders need a trusting and empathetic approach even more than purely somatic emergencies [21]. Consequently, it is often assumed that the prehospital care of a psychiatric emergency mission takes longer than, for example, the care of a trauma patient [2, 4].

Emergency physicians have a special role to play, as they are often the first to encounter patients in the acute onset of their mental disorder. Verbal interaction in the form of a “talk-down” approach may be a preventative measure against escalation in prehospital psychiatric emergencies [3, 22].

### Definition of “on-scene” time

The term “on-scene" time is well established in health services research. It is defined as the difference between the arrival of the prehospital emergency physician at the scene and the start of transport to the hospital or, in the case of outpatient care, the end of treatment [20]. During this period, medical treatment is provided with the aim of establishing transportability and further measures, such as the selection of an appropriate hospital, are planned. Patients who are in a physically and mentally stable condition do not necessarily need to be accompanied to the hospital by the emergency physician. As a medical quality criterion, the duration of the “on-scene" time plays an important role, for example in the case of polytraumatized patients. The goal is to get the patient to the hospital within the first hour after the trauma, the so-called “golden hour" of shock. This significantly increases the patient’s chances of survival [21]. There is currently no evidence regarding the optimal prehospital treatment time for psychiatric emergencies. Therefore, it is unclear whether a longer or shorter treatment time has an impact on subsequent outcome. Using data from the German Federal Highway Research Institute on the performance of emergency services in 2020/21, the average “on-scene" time for all emergencies with a dispatched emergency physician is 24 minutes. The average “on-scene" time increases to 31 minutes for critical conditions [6]. Assuming that psychiatric interventions take longer on average than interventions for patients with somatic disorders, the first step is a purely quantitative analysis of “on-scene" time. Hypothesizing that the medical behavior of male and female prehospital emergency physicians differs when dealing with prehospital psychiatric emergency patients, quantitative differences in “on-scene" time should also exist.

### Definition of “escalation of interventions”

An escalation of the prehospital psychiatric emergency was considered to have occurred if a patient had to be admitted to a psychiatric hospital with the help of the police and/or the use of restraints (involuntary admission). The administration of intravenous sedative or tranquilizing or antipsychotic drugs (propofol, haloperidol, benzodiazepines) was declared as “intravenous hypnotics” and was also defined as an escalation criterion. Involuntary admission, as defined above, combined with the administration of an intravenous hypnotic was defined as the maximum level of escalation associated with the prehospital psychiatric emergency intervention.

In general, any use of physical restraint against psychiatric patients should be avoided and is legitimate only as a last resort. The goal of any intervention must be de-escalation and the avoidance of action against the will of the mentally ill [2, 8, 23]. Escalating an intervention and deliberately inducing patient behavior that ultimately requires restraint can be considered the worst possible medical practice in prehospital care of psychiatric emergencies. In particular, the forced medication described above represents a significant violation of the integrity of patient autonomy and appears unjustified in some cases [2, 3]. Under the assumption that female emergency physicians are better able than their male colleagues to use de-escalation strategies and to avoid escalation of the intervention, the form of intervention escalation described above was examined in relation to the gender of the emergency physician. If differences are found, these could be taken into account in the education and training of emergency physicians in order to provide more appropriate prehospital care for psychiatric patients in the future.

### Definition of “emergency physician interventions”

The following emergency physician interventions were extracted from the data set and grouped according to the gender of the emergency physician: (a) no intervention performed, (b) request for crisis intervention, (c) administration of antidote (Flumazenile, Naloxone), (d) administration of oxygen or measures to secure the airway, (e) sedation measures, (f) measures to maintain cardiovascular function, (g) general monitoring (ECG, pulse oximetry, blood pressure, body temperature).

The evaluation of the measures taken should help to assess how often medical measures were taken in the prehospital setting for different psychiatric disorders. In certain conditions, such as psychiatric emergencies, it seems to make little sense to take vital signs against the patient’s will. The emergency physician is caught between the need for somatic clarification of psychiatric illnesses, the so-called “medical clearing”, also in the prehospital setting, which is generally defined as a medical duty of care, and the situationally conscious decision to refrain from measures that are initially not considered necessary. From the patient’s point of view, conscious avoidance may initially seem helpful and de-escalating. It is conceivable that there are gender differences between prehospital emergency physicians in the use or avoidance of prehospital interventions. These could be taken into account in future evaluations of treatment approaches for specific psychiatric disorders in the prehospital setting.

## Methods

### Study design and setting

We performed a monocentric, retrospective cohort analysis of prehospital emergencies with a suspected psychiatric diagnosis between January 1, 2015 and December 31, 2021 at the three ground-based rescue services of the Department of Emergency Medicine, Department of Anesthesiology and Intensive Care Medicine, University Hospital Ulm, Germany. Only anesthetists performed the evaluated missions. Data analysis was performed after approval by the Ethics Committee of the University of Ulm (Application ID: 110/22, 23.05.2022) and prospective registration in the German Clinical Trials Register (DRKS-ID: DRKS00031237 on 06.02.2023) between 07.02.2023 and 01.02.2024 at the Department of Anesthesiology and Intensive Care Medicine and the Department of Psychiatry and Psychotherapy III of the University Hospital Ulm. According to the guidelines of the reviewing ethics committee, patient information was not required due to the retrospective data analysis. Therefore, no written or verbal informed consent was obtained from patients for this study.

### Study population

The underlying data is a retrospective dataset (2015–2021) of all prehospital emergency missions handled by the prehospital emergency physicians of the Department of Anesthesiology and Intensive Care Medicine at the University Hospital Ulm, Germany.

All emergency physician records with a suspected psychiatric emergency physician diagnosis were included. The suspected diagnosis was handwritten by the emergency physician in the dispatch documentation and cannot necessarily be assigned to an ICD/DSM diagnosis.

Patients with suspected withdrawal delirium or a suspected diagnosis of delirium of another origin were excluded. The reason for this restriction was that delirium, as a syndrome complex, is not treated primarily in a psychiatric department, and belong to the syndromes that should initially be treated in intensive care units [24]. Patients with suspected dementia syndrome were also excluded, as dementia is generally not an acute medical-psychiatric condition.

### Analysis of paper-based mission documentation

The prehospital emergency physicians documented the mission using an ePen-based approach (Takwa^®^ digital pen) on templates using the MIND data set (Minimum Emergency Data Set EPRO-5.1-ABCDE [25]).

The analysis of the stored protocols was based on the recommendations of Gilbert and Lowenstein for the evaluation of chart reviews in emergency medicine research [26, 27]. The graduate student (CS) responsible for data extraction was specially trained and supervised in the analysis of the protocols by the principal investigators (BS and CSL), who were experienced in the evaluation of patient records. Protocols with inconclusive allocation were reviewed by one of the principal investigators and a final allocation or exclusion from the study was made. The stored protocols were analyzed for each index year as follows: First, all records of patients treated by prehospital emergency physicians during the study period were retrieved by the principal investigators. The selection was limited and double-checked. Only cases in which the prehospital emergency physician had documented a suspected psychiatric diagnosis were included. A detailed list of all the date extracted from these patient report forms is provided in the Supplement (Supplementary Table 1). To examine the influence of emergency physician gender on the prehospital care of psychiatric patients, the identified cases were divided into the following six subgroups according to ICD-10 and DSM 5 criteria by the graduate student and one of the principal investigators: anxiety and panic disorders, agitation, intoxication, exceptional mental situation, suicidal behavior and “psychiatric miscellaneous” (psychosocial crisis, acute stress reaction). “Exceptional mental situation” can be checked in the EPRO-5.1-ABCDE protocol following the item “psychiatric”. The patient is assigned purely descriptively, e.g. if the patient is in an exceptional mental situation due to an accident, a psychosocially stressful event, etc., but does not meet the DSM 5 criteria for an acute stress reaction. The analyzed prehospital psychiatric emergencies were differentiated according to the gender of the emergency physician (male/female).

“On-scene" time is defined as the difference between the arrival of the prehospital emergency physician at the scene and the start of transport to the hospital or, in the case of outpatient care, the end of treatment. All data analyzed in the study refer to the “on-scene" time and can be clearly assigned to this period in the retrospective data set.

### Statistical analysis

First, a descriptive analysis of the variables was performed. Arithmetic mean, standard deviation, minimum and maximum were used to describe metrically scaled data. Absolute and relative frequencies were used to describe categorical data.

We used the chi-squared test for comparisons and subgroup analyses; and the Mann-Whitney U-test for non-normally distributed metrically scaled data for categorical characteristics. Because of the nonrandomized data structure and the associated risk of biased estimates due to confounding, the inferential statistical analyses were accompanied by appropriate univariate and multivariate regression models to determine the stability of the results. We used linear regression for continuous outcomes and binary logistic regression for dichotomous outcomes. The significance level was set at *p* < 0.05. Analysis and evaluation were performed using Microsoft Excel and IBM SPSS Statistics, version 29.

## Results

**Fig. 1:**
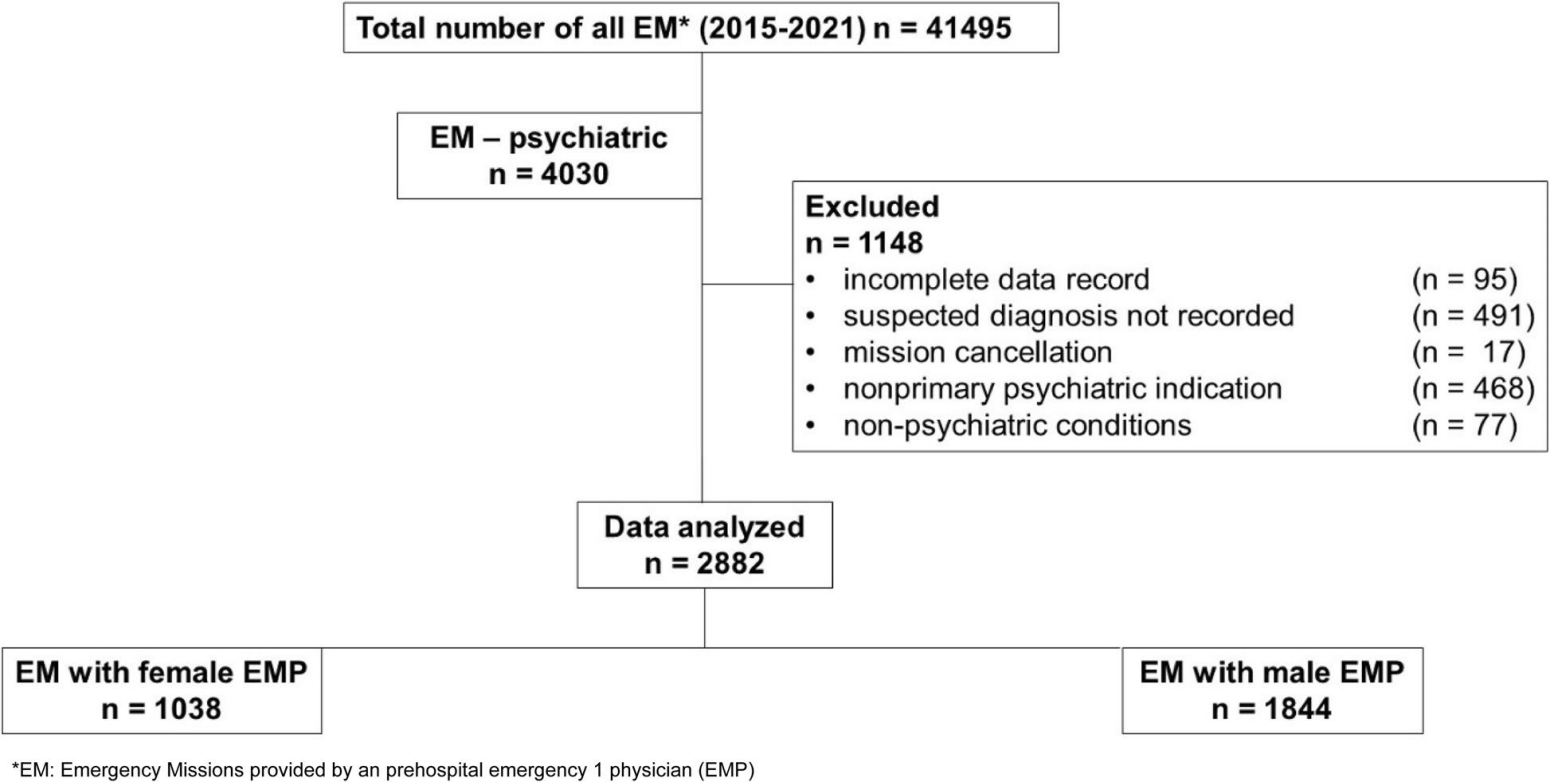
Study flowchart

**Fig. 2:**
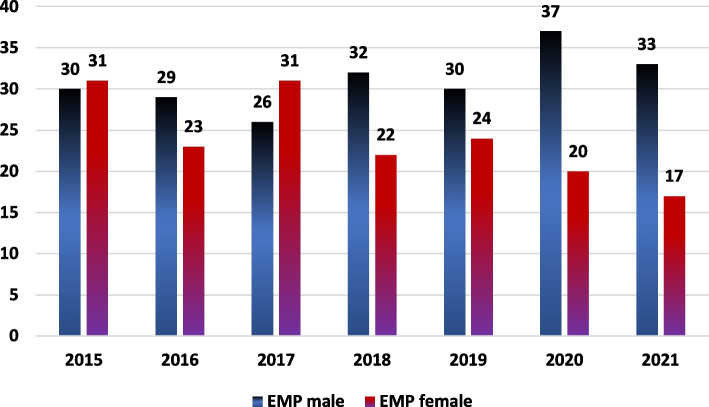
Number of emergency physicians during the reporting period. Male EMPs (Emergency Physicians) are shown in blue bars, and female EMPs are shown in red bars. Absolute values are shown as numbers above the columns, separated by gender. EMP: Emergency Physician

As shown in Fig. 1, a total of 2882 emergency missions with a psychiatric indication/prehospital psychiatric diagnosis were studied. On average, 31 male (STD 3.5) and 24 female (STD 5.3) emergency physicians, as stated in the EPRO 3.1 protocol, were dispatched during the study period. Figure 2 shows the corresponding graph of emergency physicians in duty, differentiated by gender and year.

Among the prehospital psychiatric emergencies, 56% (*n* = 1606) were male and the median age was 38 years (male 38, STD 16.8, female 38.8, STD 21.7).

According to the subgroups mentioned above, the prehospital psychiatric emergencies were divided as follows: intoxication (*n* = 1343, 46.6%), suicidal behavior (*n* = 488, 16.9%), exceptional mental situation (*n* = 282, 9.8%), agitation (*n* = 262, 9.1%), anxiety and panic disorders (*n* = 262, 9.1%) and “psychiatric miscellaneous” (*n* = 245, 8.5%).

Inpatient hospitalization occurred in 67.9% (*n* = 1958) of prehospital psychiatric emergency cases. Of these, 20.3% (*n* = 392) were admitted directly to a psychiatric hospital.

### Effect of emergency physician gender on the “on-scene” time in prehospital psychiatric emergencies

As shown in Table 1, male emergency physicians spend slightly more time with patients with anxiety and panic disorders than their female colleagues (*p* = 0.024). Female emergency physicians show significantly greater variation in "on-scene" time compared to male emergency physicians (*p* = 0.025). An overview of the patient demographics (age, gender distribution) can be found in the supplement (Supplementary Table 2).

**Table 1:** "On-scene" time in minutes (median and interquartile range) for prehospital psychiatric emergencies, differentiated by female and male emergency physicians and grouped into the six most common entities. EMP: Emergency Physician, EM: Emergency Mission, IQR: Interquartile Range

	Female EMPs (*n* = 24)		Male EMPs (*n* = 31)		*P* value
	EM (*n*)	EM – median duration of on-scene time (IQR)	EM (*n*)	EM – median duration of on-scene time (IQR)	
**Intoxications**	380	19 [13.0]	963	20 [12.0]	0.05
**Agitation**	155	28 [18.5]	107	28 [18.0]	0.845
**Suicidal behavior**	162	23 [13.3]	326	24 [14.0]	0.462
**Exceptional mental situation**	196	23 [15.0]	86	22 [13.0]	0.963
**Anxiety and panic disorder**	177	22 [11.5]	85	23 [11.0]	0.024
**Psychiatric miscellaneous**	121	23 [14.0]	124	22.5 [14.0]	0.798
**Overall**		**22 (14.0)**		**22.0 (13.0)**	**0.025**

### Effect of emergency physician gender on escalation of interventions for patients with a suspected psychiatric diagnosis

**Table 2:** Escalation of interventions in prehospital psychiatric emergencies, differentiated by female and male emergency physicians. Figures in absolute numbers (n) and percentages (in brackets) in relation to the population. EMP: Emergency Physician, EM: emergency mission

	Female EMPs (*n* = 24)	Male EMPs (*n* = 31)	*P* value
	Number of EMs *n* = 1038	Number of EMs *n* = 1844	
Intravenous hypnotics – Administered by EMP	12 (1.2%)	57 (3.1%)	0.001
Involuntary Psychiatric Admission – Forced by EMP	33 (3.2%)	67 (3.6%)	0.522
Involuntary Psychiatric Admission and administration of intravenous hypnotics as a last resort by EMP	45 (4.3%)	124 (6.7%)	0.009

As a surrogate for an escalation of prehospital care for prehospital psychiatric emergency, the study evaluated the administration of intravenous hypnotics. Depicted in Table 2, significantly more male emergency physicians administered intravenous hypnotics in psychiatric emergencies than their female counterparts (*p* = 0.001). For psychiatric patients who refused medically indicated inpatient psychiatric admission (“involuntary psychiatric admission”), male and female emergency physicians were equally likely to take the required action (notification of the police and, if necessary, the use of coercive police measures, *p* = 0.522). As defined above, the maximum level of escalation, i.e. the combination of involuntary admission with the use of intravenous hypnotics, was performed significantly more often by male emergency physicians (*p* = 0.009).

Subgroup analysis of the frequency of medication administration or lack thereof suggested that the gender of the emergency physician had no significant effect in the population studied (Supplementary Table 3).

### Analysis of emergency physician intervention

 Emergency physician interventions, as described in the methods, in each of the subgroups, female prehospital emergency physicians were significantly less likely than their male counterparts to insert an intravenous catheter in patients with anxiety or panic disorders (*p* = 0.042). For patients with psychosocial crisis or acute stress reactions (psychiatric miscellaneous), female prehospital emergency physicians were significantly more likely to refrain from taking any action (*p* = 0.042) (Table 4 Supplement). However, a professional crisis intervention team was more likely to be requested in psychiatric emergency missions managed by male emergency physicians (*p* = 0.021) (Supplementary Fig. 1).

## Discussion

This is the first study analyzing the influence of emergency physician gender on prehospital care of psychiatric emergencies. Although there are no relevant gender differences in “on-scene” times for prehospital care of psychiatric emergencies, male physicians administered intravenous hypnotics significantly more often than their female counterparts whereas female physicians were more likely to refrain from invasive measures, especially in patients with psychosocial crisis or acute stress reaction.

### Influence of physician gender on “on-scene" time

When emergency physicians are asked about the factors that distinguish prehospital psychiatric emergencies from the care of other patient populations, the time invested in exploration and consent for further procedures (commitment) are often cited as eminent factors [4, 28]. However, valid data to objectify this opinion is still lacking [2]. Our study did not reveal an association between prehospital physician gender and “on-scene" time in prehospital psychiatric emergencies. Comparing the “on-scene" times of the present study with the German national data, the overall times are shorter. The subjective and sometimes generalizing assessment of emergency physicians that psychiatric emergencies take longer seems to be refuted.

One of the possible reasons for a shorter “on-scene" time is incorrect scheduling of the prehospital emergency physician, which, according to the German Medical Association, occurs in up to 30% of all emergency physician deployments [29]. If paramedics are already at the psychiatric emergency, it is possible that by the time the prehospital emergency physician arrives, the situation will have calmed down and there will no longer be a need for direct medical intervention. Sometimes, medical-legal reasons, such as the decision to use involuntary hospitalization, also lead to the prehospital emergency physician being called [2, 3, 21, 23, 30].

### Influence of physician gender on escalation of care and prehospital interventions

The general willingness of the prehospital emergency physicians in our study, all of whom are board-certified anesthesiologists or anesthesiology residents, to use intravenous hypnotics in prehospital psychiatric emergencies is consistent with a prospective survey of emergency physicians who also exclusively practiced anesthesiology [3]. In principle, the use of a sedative, for example in cases of severe agitation, is an effective treatment in the sense of a so-called rapid tranquilization [31, 32]. However, the data do not reveal why male emergency physicians were more likely to choose intravenous hypnotics. One possible explanation is the gender difference in decision making between male and female physicians observed by Gotlieb et al. One of the findings was that male physicians used a more heuristic approach, while female doctors relied more on available information [33]. “Heuristic approach” means that the physician makes decisions or conclusions based on limited information. The resulting action is usually different from an optimal solution.

Mission escalation is rarely monocausal. Whittington and Wykes have defined the concept of “aversive stimulation” as a behavior that can provoke fear and anger in inpatients with psychiatric disorders [34]. It is conceivable, for example, that the threat of forced medication, a request for hospitalization, or even an aggressive communication style in the event of a psychiatric emergency in the prehospital setting may also increase the likelihood of violence. In this context, the conscious decision to refrain from medical diagnostic measures in the acute situation seems justified, especially in the case of agitation [2, 8, 23]. The fact that female prehospital emergency physicians were less likely to use invasive measures for the treatment of anxiety and panic disorders, and less likely to use further measures for acute stress reactions, suggests a different treatment approach compared to male emergency physicians. A retrospective study published in 2024 by Miyawaki et al. showed that treatment by female physicians can have a positive impact on mortality and readmission rates [35]. The study group led by Roter et al. postulates that female physicians “engage in significantly more active partnership behaviors, positive talk, psychosocial counseling, psychosocial question asking, and emotionally focused talk” [14, 36]. Finally, Christen et al. get to the heart of the matter with their statement “it is not the gender of the physician, but the gender-specific communication skills, especially patient-centered communication,” which is the critical factor [37].

### Approaches for improvement of prehospital care of psychiatric emergencies

The aim of this study was to generate ideas for improving the education and training of prehospital emergency physicians from a gender perspective. In line with the comprehensive training of in-hospital emergency physicians which is common in many countries, the authors recommend the professionalization of prehospital emergency medicine [38–40]. Due to the high prevalence of psychiatric emergencies in the prehospital setting, a mandatory rotation at an inpatient psychiatric facility might be reasonable. An interesting approach for further studies would be a video-based analysis of the performance of prehospital emergency physicians in order to consider gender-specific effects, such as gender-specific communication behavior, in future education and training [41, 42].

### Limitations

The study has some limitations that need to be addressed. First, documentation quality of the prehospital missions was deficient in a relevant number of cases, which had to be excluded from further analysis. Second, the selection of a patient population treated exclusively by prehospital emergency physicians, Anesthesia residents and specialists only, may lead to a selection bias compared to a data set that would also include emergency missions performed by paramedics; this should be considered in further studies. Third, the chosen research question may run the risk of considering purely gender-specific stereotypes.

## Conclusion

Psychiatric emergencies require the emergency physician to develop a nuanced approach that moves away from algorithms or standardized pathways of care. As might be expected given the extensive literature on gender differences, the retrospective analysis did not provide clear evidence of clinically relevant, i.e., in terms of potential influence on daily patient care, gender differences in the prehospital care of psychiatric emergencies. Studies that differentiate between biological sex, gender, and individual personality aspects are needed to further address this issue.

## Supplementary Information

Supplementary Material 1.

## Data Availability

The data sets created and/or analyzed as part of this study are not publicly available, as some information is used for internal quality analysis only. All other results and data collected are reported entirely within the scope of this manuscript. If there is a justified interest in accessing it, the data can be requested from the corresponding author.

## Abbreviations

EM: Emergency Mission
EMP: Emergency Physician

## Glossary

Emergency physician interventions: Prehospital emergency physician interventions were defined as prehospital medical interventions included in the MIND (Minimum Emergency Data Set EPRO-5.1-ABCDE) dataset, such as "no intervention performed", “request for crisis intervention”, “administration of antidote (Flumazenile, Naloxone)” “administration of oxygen or measures to secure the airway”, “sedation measures”, “measures to maintain cardiovascular function”, “general monitoring (ECG, pulse oximetry, blood pressure, temperature)”.
Escalation of interventions: An escalation of the prehospital psychiatric emergency was considered to have occurred if a patient had to be admitted to a psychiatric hospital with the help of the police and/or the use of restraints (involuntary admission). The administration of intravenous sedative or tranquilizing or antipsychotic drugs was declared as "intravenous hypnotics" and was also defined as an escalation criterion. Involuntary admission combined with the administration of an intravenous hypnotic was defined as the maximum level of escalation associated with the prehospital psychiatric emergency intervention.
"On-scene" time: The prehospital duration of the psychiatric emergency operation was calculated from the time the emergency physician arrived at the patient to the time the ambulance departed, as physician-led transport was not always provided.
