# Supplementary material for: Does the gender of emergency physicians have an impact on the prehospital care of psychiatric emergencies? a retrospective cohort analysis
Source: BMC Emerg Med. 2024 Oct 24;24:201. doi: 10.1186/s12873-024-01118-3 (PMC11515376; doi:10.1186/s12873-024-01118-3)
Supplement: Supplementary file 1 — Supplementary Material 1. [file 12873_2024_1118_MOESM1_ESM.docx]

**Does the gender of emergency physicians have an impact on the prehospital care of psychiatric emergencies? A retrospective cohort analysis.**

**Supplement**

[Benedikt Schick](https://pubmed.ncbi.nlm.nih.gov/?term=Schick%20B%5BAuthor%5D),^🖂1^ [Benjamin Mayer](https://pubmed.ncbi.nlm.nih.gov/?term=Mayer%20B%5BAuthor%5D),^2^ [Bettina Jungwirth](https://pubmed.ncbi.nlm.nih.gov/?term=Jungwirth%20B%5BAuthor%5D),^1^ [Eberhard Barth](https://pubmed.ncbi.nlm.nih.gov/?term=Barth%20E%5BAuthor%5D),^1^ [Claus-Martin Muth](https://pubmed.ncbi.nlm.nih.gov/?term=Muth%20CM%5BAuthor%5D),^1^ Christine Eimer³, Celine Schwarzer^4^ and [Carlos Schönfeldt-Lecuona](https://pubmed.ncbi.nlm.nih.gov/?term=Sch%C3%B6nfeldt-Lecuona%20C%5BAuthor%5D)^4^

^1^Department of Anesthesiology and Intensive Care Medicine, University Hospital Ulm, Albert-Einstein-Allee 23, 89081 Ulm, Germany

^2^Institute of Epidemiology and Medical Biometry, Ulm University, Schwabstraße 13, 89075 Ulm, Germany

^³^Department of Anesthesiology and Intensive Care Medicine, University Medical Center Schleswig-Holstein, Campus Kiel, Arnold-Heller Str. 3, 24105 Kiel, Germany

^4^Department of Psychiatry and Psychotherapy III, University Hospital Ulm, Leimgrubenweg 12–14, 89075 Ulm, Germany

Benedikt Schick, E-mail: benedikt.schick@uni-ulm.de

^🖂^Corresponding author.

**Table of contents**

Supplementary table 1 – Overview of all data extracted from MIND protocols

Supplementary table 2 – Overview of the patient demographics

Supplementary figure 1: Overview of prehospital emergency medical care for psychiatric emergencies

Supplementary table 3: Overview of the administered Medication in the psychiatric EM by the EMP

Supplementary table 4: Overview of all measures according to the subgroups of psychiatric EM

**Supplementary table 1** – Overview of all data extracted from MIND protocols

| **Variable** | **Definition** |
| --- | --- |
| Alarm times | Patient arrival to ambulance departure |
| Name of EMP | Secondary inference about sex |
| Date of intervention | Year and Month |
| Patients age and sex | Years/ male-female |
| Findings in the psychiatric section of the protocol | - anxious - suicidal - slowed down - euphoric - motor restless - aggressive - delusional - agitated - depressed - confused |
| Suspected psychiatric diagnosis | - Psychological Emergency - Psychosocial crisis - Depression - Mania - other - Intoxication (alcohol, drugs, medications, other) - Withdrawal/delirium - Suicidal tendencies   or a free text diagnosis by the EMP |
| Transport destination | - No transport - Admission to psychiatric hospital - Admission to hospital/Emergency Department |
| Type of admission and accommodation* | - No inpatient psychiatric admission - Inpatient psychiatric admission with informed consent - Inpatient psychiatric admission without informed consent - with police support - with use of restraints |
| EMPs treatment | - Medically administered sedation (antipsychotics, benzodiazepines, hypnotics such as propofol) - Administration of an antidote (flumazenil - benzodiazepine intoxication, naloxone - opiate intoxication) - Airway management (oxygen administration, airway clearing, suctioning, intubation if necessary) - Circulation (especially intravenous access, administration of crystalloid infusion up to extended interventions according to ERC guidelines) - Monitoring (ECG/12-lead ECG, temperature, non-invasive blood pressure, SpO2, blood glucose) - Requesting crisis intervention |
| Supplementary table 1 provides an overview of all data extracted from MIND protocols (Minimum Emergency Data Sets).  * By analyzing the psychiatrist's letters for each case, it was possible to determine retrospectively whether the patient was admitted involuntarily or with the help of the police using coercive measures. EMP: Emergency Physician | |

**Supplementary table 2 – Overview of the patient demographics**

|  | **Female Patients**  **(n = 1039)** | **Male Patients**  **(n = 1845)** | **P value** |
| --- | --- | --- | --- |
| **Intoxications** | | | |
| Age* | 32.5 (19.5) | 37.4 (15) | <0.001 |
| Sex, n (%) | 380 (36.6) | 963 (52.2) | <0.001 |
| **Agitation** | | | |
| Age* | 41.3 (23.4) | 40.2 (21.2) | 0.761 |
| Sex, n (%) | 155 (14.9) | 107 (5.8) | <0.001 |
| **Suicidal behavior** | | | |
| Age* | 41.7 (20.4) | 41.1 (18.4) | 0.873 |
| Sex, n (%) | 162 (15.6) | 326 (17.7) | 0.227 |
| **Exceptional mental situation** | | | |
| Age* | 35.7 (21.3) | 39.2 (18.8) | 0.351 |
| Sex, n (%) | 196 (18.9) | 86 (4.7) | <0.001 |
| **Anxiety and panic disorder** | | | |
| Age* | 51.6 (22.8) | 41.0 (19.0) | 0.005 |
| Sex, n (%) | 177 (17.0) | 85 (4.6) | <0.001 |
| **Psychiatric miscellaneous** | | | |
| Age* | 46.9 (23.3) | 39.4 (19.0) | 0.042 |
| Sex, n (%) | 121 (11.6) | 124 (6.7) | <0.001 |

Overview of the age and gender distribution of psychiatric emergency patients, differentiated by subgroups. *Age is given as median with standard deviation. The gender distribution in absolute values and as a percentage of the total number of psychiatric emergency patients.

**Supplementary figure 1:** Overview of prehospital emergency medical care for psychiatric emergencies. A distinction is made between male emergency physicians (♂: broad shaded bars) and female emergency physicians (♀: narrow shaded bars).

| **Supplementary table 3:** Overview of the administered Medication in the psychiatric EM by the EMP | | | | | | | | | | | | |  |
| --- | --- | --- | --- | --- | --- | --- | --- | --- | --- | --- | --- | --- | --- |
| EM (n) | | Antipsychotics  n (%) | | P value | Benzodiazepines  n (%) | | P value | Multiple dosing  n (%) | | P value | No need for psychiatric medi-cation n (%) | | P value |
| ♀ | ♂ | ♀ | ♂ |  | ♀ | ♂ |  | ♀ | ♂ |  | ♀ | ♂ |  |
| **Intoxications** | | | | | | | | | | | | | |
| 500 | 843 | 2  (0.4) | 2  (0.2) | 0.99 | 31 (6.2) | 55  (6.5) | 0.88 | 4  (0.8) | 2 (0.2) | 0.29 | 461  (92.2) | 773 (91.7) | 0.98 |
| **Agitation** | | | | | | | | | | | | | |
| 97 | 165 | 2  (2.1) | 2  (1.2) | 0.99 | 33 (34.0) | 50 (30.3) | 0.75 | 6  (6.2) | 7 (4.2) | 0.71 | 57 (58.8) | 102  (61.8) | 0.89 |
| **Suicidal behavior** | | | | | | | | | | | | | |
| 162 | 326 | -- | 2  (0.6) | -- | 12 (7.4) | 35 (10.7) | 0.36 | 1  (0.6) | -- | -- | 148 (91.4) | 285 (87.4) | 0.81 |
| **Exceptional mental situation** | | | | | | | | | | | | | |
| 113 | 169 | -- | -- | -- | 35 (31.0) | 46 (27.2) | 0.71 | -- | 1 (0.6) | -- | 78 (69.0) | 121 (71.6) | 0.92 |
| **Anxiety and panic disorder** | | | | | | | | | | | | | |
| 89 | 173 | -- | -- | -- | 42 (47.2) | 77 (44.5) | 0.89 | 2  (2.3) | 3 (1.7) | 0.85 | 45  (50.6) | 90 (52.0) | 0.99 |
| **Psychiatric miscellaneous** | | | | | | | | | | | | | |
| 77 | 168 | -- | -- | -- | 9 (11.7) | 19  (11.3) | 0.89 | 2  (2.6) | 2 (1.2) | 0.80 | 66 (85.7) | 144 (85.7) | 0.92 |

Supplementary table 3 provides an overview of the medications administered by the EMP in psychiatric EMs in relation to the sex of the EMP. A distinction is made between the administration of an antipsychotic, the administration of a benzodiazepine, and the repeated administration of one of these drugs. If no medication was administered, this was also indicated. “Psychiatric miscellaneous“ summarizes psychosocial crisis and acute stress reactions. EMP: Emergency Medical Physician, EM: Emergency Mission, ♀: female EMP, ♂: male EMP, -- corresponds to n=0 (0%).

**Supplementary table 4: Overview of all measures according to the subgroups of psychiatric EM**

| **Variable** | **Intoxication** | | **P value** | **Agitation** | | | **P value** | **Suicidal behavior** | | **P value** | **Exceptional mental situation** | | **P Value** |
| --- | --- | --- | --- | --- | --- | --- | --- | --- | --- | --- | --- | --- | --- |
|  | EMP (n (%)) | |  | EMP (n (%)) | | |  | EMP (n (%)) | |  | EMP (n (%)) | |  |
|  | ♀ | ♂ |  | ♀ | | ♂ |  | ♀ | ♂ |  | ♀ | ♂ |  |
|  | 500 | 843 |  | 97 | | 165 |  | 162 | 326 |  | 113 | 169 |  |
| **Sedation** | 41 (8.2) | 76 (9.0) | 0.711 | 41  (42.3) | | 63 | 0.758 | 17  (10.5) | 43  (13.2) | 0.540 | 35  (31.0) | 48  (28.4) | 0.830 |
| **Antidote** | 64 (12.8) | 96  (11.4) | 0.550 | -- | | -- | -- | 4  (2.5) | 3  (0.9) | 0.353 | -- | -- | -- |
| **Airway** | 131  (26.2) | 221  (26.2) | 0.955 | 3  (3.1) | | 15  (9.1) | 0.136 | 13  (8.0) | 46  (14.1) | 0.112 | 1  (0.9) | 8  (4.7) | 0.160 |
| **Circulation** | 275  (55.0) | 417  (49.5) | 0.291 | 17  (17.5) | | 44  (26.7) | 0.231 | 61  (37.6) | 125  (38.3) | 0.994 | 25  (22.1) | 47  (27.8) | 0.487 |
| **Monitoring** | 468  (93.6) | 786  (93.2) | 0.994 | 67  (69.1) | | 127  (77.0) | 0.654 | 113  (69.7) | 240  (73.6) | 0.774 | 92  (81.4) | 144  (85.2) | 0.871 |
| **No measures carried out** | 30  (6.0) | 57  (6.8) | 0.689 | 30  (30.9) | | 36  (21.8) | 0.265 | 49  (30.2) | 86  (26.4) | 0.568 | 21  (18.6) | 25  (14.8) | 0.581 |
| **Crisis intervention** | 7  (1.4) | 23  (2.7) | 0.171 | 2  (2.1) | | 11  (6.7) | 0.197 | 15  (9.3) | 24  (7.4) | 0.621 | 8  (7.1) | 20  11.8) | 0.324 |
|  | **Axiety and panic disorder** | | **P value** | **Psychiatric miscallenous** | | **P value** |  | | | | | | |
|  | EMP (n (%)) | |  | EMP (n (%)) | |  |  |  |  |  |  |  |  |
|  | ♀ | **♂** |  | ♀ | **♂** |  |  |  |  |  |  |  |  |
|  | 89 | 173 |  | 77 | 168 |  |  |  |  |  |  |  |  |
| **Sedation** | 44  (49.4) | 84  (48.5) | 0.973 | 11  (14.3) | 25  (14.9) | 0.931 |  |  |  |  |  |  |  |
| **Antidote** | -- | -- | -- | 1  (1.3) | 3  (1.8) | 0.788 |  |  |  |  |  |  |  |
| **Airway** | 4  (4.5) | 8  (4.6) | 0.790 | 10  (13.0) | 22  (13.1) | 0.856 |  |  |  |  |  |  |  |
| **Circulation** | 14  (15.7) | 55  (31.8) | **0.042** | 23  (29.9) | 64  (38.1) | 0.462 |  |  |  |  |  |  |  |
| **Monitoring** | 83  (93.3) | 167  (96.5) | 0.928 | 54  (70.1) | 141  (83.9) | 0.455 |  |  |  |  |  |  |  |
| **No measures carried out** | 6  (6.7) | 6  (3.5) | 0.406 | 23  (29.9) | 25  (14.9) | **0.042** |  |  |  |  |  |  |  |
| **Crisis intervention** | 4  (4.5) | 14  (8.1) | 0.444 | 1  (1.3) | 9  (5.4) | 0.275 |  |  |  |  |  |  |  |

Supplementary table 4 provides an overview of all measures according to the subgroups of psychiatric emergencies. The variables refer to the six defined subgroups. EMP: Emergency Medical Physician, EM: Emergency Mission, ♀: female EMP, ♂: male EMP, -- corresponds to n=0 (0%).
